# Gynecology and Obstetrics

**Published:** 1894-01

**Authors:** 


### GYNECOLOGY AND OBSTETRICS.

EDITED BY DR. VIRGIL O. HARDON.

### THE INFLUENCE OF TREATMENT OF A SYPHILITIC MOTHER, ESPECIALLY DURING PREGNANCY, UPON THE HEALTH OF THE INFANT.

Etienne (*Annales de Gyn.*, April, 1892; *Am. Journal of Obstet.*, February, 1893) draws the following conclusions from the study of thirty-two cases of pregnancy in syphilitic women:

1. The fetal mortality, in cases in which the mother has never been treated, is enormous, reaching 95.5 per cent. If treatment be applied throughout pregnancy, we may hope to obtain almost complete immunity from this infant mortality.

2. Syphilis attacks the fetus especially during the fifth, sixth and seventh months of intrauterine pregnancy.

3. Paternal syphilis is less injurious to the fetus than maternal syphilis.

4. The prognosis differs according to the stage of pregnancy when the mother becomes contaminated.

(*a*) If infection occurs within the first three months and is not treated, the mortality during the first few days after delivery reaches one hundred per cent.

(*b*) The prognosis is a trifle better if infection occurs during the fourth and fifth months.

(*c*) In one case of infection at the eighth month, the child lived and was apparently healthy; in a second similar case the child was in good condition at birth, but developed symptoms of syphilis later.

(*d*) Whenever suitable treatment was administered the mortality was *nil*.

5. In no case were any unfavorable results met with from internal medication during pregnancy.—*Boston Med. and Surg. Jour.*

---

### CRANIECTOMY.

At the Paris Academy of Medicine Dr. Bourneville read a paper upon the above subject, which may be resumed as follows:

The examination of 350 crania collected in the Bicetre Hospital, proves that, in idiotic and backward children, premature synostosis of the sutures does not generally exist.

Inasmuch as the exact state of the sutures and the thickness of the cranium cannot be determined with any great degree of accuracy in the living, it must follow that craniectomy must be done without exact data, and frequently upon crania which are perfectly able to give the brain all the space it needs.

The examination of these crania shows, also, that craniectomy is not able to give the brain any very great degree of expansion. These facts justify the statement of anthropologists who assert "that the brain makes the cranium, and moulds it upon itself." Besides this, the encephalic lesions, to which are due the greater number of the forms of idiocy, are deep, varied and extensive, hence not susceptible of being modified by craniectomy.

Most of the surgeons who have practiced this operation agree upon the fact that in most instances their operative measures have been followed by slight or doubtful results, or by no improvement whatever; that serious accidents (paralysis, convulsions, etc.), and even death (13 times in 85 operations collected by the author), have resulted from it.—*Semaine Médicale.*

---

### CONCERNING THE ETIOLOGY AND TREATMENT OF PREMATURE LABORS AND ABORTIONS.

Stumpff (*Münchener medicinische Wochenschrift*, 1892, Nos. 43 and 44) quotes from Winckel's results in the conservative treatment of abortion and premature labor in the Münchener Frauen Klinik from 1885 to 1892.

In five and a half years there were treated 184 abortions, 115 cases of immature labor, and 147 cases of premature labor. The first two occurred in mothers from 26 to 30 years of age, and the last in mothers from 21 to 25 years old. The least possibility of the interruption of labor was before the 25th year, the greatest after the 40th year. In primiparæ the danger increased with the advance in the months of pregnancy; with multiparæ the converse. One-third of all interruptions of labor occurred in the third month; 50 per cent. of all aborted ova were expelled *in toto*. Etiologically, preceding interruptions, as manual placental separation, are of much importance. 41.7 per cent. occurred from uterine diseases; in 56.2 per cent. there were affections on the part of the mother; in 29.5 per cent. in the fetal membranes; and in 14 per cent. in the fetus.

From 169 abortions, 84 per cent. recovered by tamponade without any disaster; 4.1 per cent. had hemorrhages following; 1.2 per cent. suffered from ergotism; 10.7 per cent. with light infection. In cases in which there was retention of the membranes the results were not quite so good, hemorrhage following three times as often. The fear of retained decidua in abortion is carried too far.

The investigations of Puppe, arranged by Winter, have shown that a uterine mucosa perfectly renewed after an abortion is in poorer condition to play the rôle of a decidua of pregnancy than one which has performed this function entirely or partly before. Klein has found that the decidua vera after abortion remains entirely or partly in the uterus; that the decidual cells and glands and superficial epithelium degenerate and become absorbed, and are then replaced through connective tissue cells and cylinder epithelium, the deeper layers remaining. The vera is transformed into mucosa in four to six weeks. Endometritis post abortum is not the result of retention of decidua, but existed previously, and was the cause of abortion. According to J. Veit, the endometritis remains in spite of curettage.

Bleeding placental pieces must be removed with the finger. Of these cases 66.7 per cent. recovered without incident; 13.3 per cent. suffered from hemorrhage; 20 per cent. from infection. Septic abortions are also to be removed by the finger, followed by the irrigation of the uterus by means of the uterine catheter, with a 3 per cent. solution of carbolic acid. The iodoform gauze tampon is also recommended.

One hundred and fifteen of the cases were of immature labor; these were less favorable. Ninety-one recovered without incident; thirteen suffered from slight fever; five developed general sepsis; in one case there was purulent parametritis; one developed thrombosis of the veins; six died. Manual placental separation was performed nine times with perfect recovery; three times with subsequent fever, and three times with fatal sepsis.

---

### THE DISORDERS OF THE NERVOUS SYSTEM ASSOCIATED WITH THE CHANGE OF LIFE.

Dr. Gustavus Eliot, in concluding this article, offers the following propositions:

1. At the time of life when the menopause occurs the various organs of a woman's body are likely to be in a state of depression as regards either their nutrition or functional activity, so that the normal equilibrium of health action may be easily disturbed, and abnormal action, the manifestation of disordered function, may be inaugurated and perpetuated.

2. The cessation of menstruation is an event of great physiological importance, and is perfectly competent to produce grave disturbances of the nervous system, if any predisposition to them already exists.

3. The more common disorders of the nervous system occurring under these circumstances are functional in character, and are associated with disturbances of functions of other organs, and especially of the digestive, circulatory and hematopoetic systems.

4. In their treatment, attention should first be paid to improving the general nutrition of all the tissues of the body, and restoring each organ to its normal activity.

5. If, after all other organs have resumed the proper performance of their functions, symptoms referable to a disordered condition of the nervous system still persist, recourse must be had to remedies which act directly upon the nervous system, either by improving its nutrition or by modifying and regulating its action.

---

### MANTON ON AFTER-TREATMENT OF CELIOTOMY CASES.

Literature has abounded in the past few years with reports of the various conditions that have led to abdominal section, and in regard to its operative *technique;* but little has been said of the after-care of the patients. The three great evils most apprehended after abdominal section are shock, hemorrhage and peritonitis.

*Shock,* which has been defined as a "general paresis of the nervous system," is rare when excessive loss of blood has been guarded against and the patient kept warm. Complete anesthesia lessens the tendency to this condition. The two most important agents in its treatment are heat and stimulants. For stimulants, the hypodermatic use of nitroglycerine, one-hundredth of a grain, and strychnia from one-fiftieth even to a quarter of a grain, are the best. Alcoholic stimulants may be administered per rectum. Ordinarily the patient reacts within two or three hours, and the dangers from shock are at an end.

*Hemorrhage.* This occurs usually within a few hours after operation, and results from careless ligation of the pedicle, its unavoidable slipping in some cases, or the soft and friable condition of the tissues. The drainage tube is valuable as a "signal" in such cases, and has enabled the surgeon by prompt measures to save his case.

*Peritonitis* is due to septic absorption, and is best treated by saline purgatives. Alcohol is valuable here, as in puerperal infection.

Pain, when not too severe, is a wholesome tonic, and is usually mitigated in twelve to twenty-four hours. Opium should be avoided, and when administered should be in the form of McMunn's Elixir, twenty or thirty drops by the rectum, or of codeine.

Vomiting occurring early is generally from the anesthetic, late—several days after the operation—is generally the precursor of peritonitis or intestinal obstruction. Where the patient gags and retches and throws up little or nothing, a small cup of plain hot tea or water may be given, that the stomach may have something to act upon. Small repeated doses of carbonated waters cleanse the stomach. When these measures fail an attempt should be made to move the bowels with saline cathartics and turpentine enemas.

*Diet.* For twenty-four hours after operation nothing, unless a small amount of plain hot tea or hot water, should be given. Then two ounces of beef tea, not milk, or broth may be given every hour, or rectal alimentation may be relied upon. On the third day the bowels are to be moved and the patient gradually led toward her regular diet. Perfect quiet on the back need not be insisted upon, but the patient may be turned on the side, or bolstered up in bed, as soon as the immediate effects of the operation have passed away. To remove gas, a syringe nozzle may be introduced, and left in rectum for periods of half an hour.—*American Lancet.*

---

### TO BEGINNERS IN LAPAROTOMY.

Dr. F. Byron Robinson offers the following practical suggestions to laparotomists:

1. Remember it is criminal to learn to do laparotomy on a patient.

2. Do not attempt to do laparotomies in private houses and with no nurses.

3. Before doing any laparotomy be sure to study under a master, and assist him, if possible, so you can see the pathology in the abdomen and how he removes it. Ask him to allow you to tie a knot once in a while. Never lose the chance of assisting in or witnessing a laparotomy.

4. Learn the after-treatment. Half the battle is with the intestines.

5. Study carefully the abdominal and pelvic viscera of the cadaver. Study as many cadavers as you can. Never lose the chance of doing a post-mortem or attending one. Study the dog's viscera.

6. Be sure to make sytematic experiments on dogs' abdominal viscera. Always do the autopsy on your dogs yourself. Note what damage to the peritoneum your manipulations did. Observe what peritonitis really is.

7. Be clean without chemicals. Learn to use very few instruments. Beginners should always invite a laparotomist friend to be present.

8. Be careful of promises.—*Medical Age.*

---

### Alumnol.

A. Kontz has used alumnol in sixteen gynecological cases: catarrh of the neck, endometritis with or without inflammation of the adnexa. Cervical catarrh and simple perimetritis are very easily cured by several treatments. Endometritis, complicated with lesions of the adnexa, does not respond to this treatment; in fact, the pains are augmented on account of the irritation produced by the alumnol. Blennorrhagic vaginitis is readily cured by this remedy. The author has made use of the following preparations: A solution of 3 per cent. for lavages; a powder and bougies of 20 per cent.; a 10 per cent. solution as an astringent in the treatment of endometritis and of erosions. Gauze imbibed in a 24 per cent. solution has also rendered good results, but care must be taken not to leave it in place more than twenty-four hours, because it then takes on a very fetid odor.—*Arch. of Gynecology.*

### THE INTRA-UTERINE TAMPON.

Andrew F. Currier (*Annals of Gynecology and Pediatry*, October, 1893) remarks that while the vaginal tampon is often an indirect and unsatisfactory means of relieving uterine trouble, the intra-uterine tampon goes directly to the source of trouble, and its employment has opened a new field in intra-uterine therapeutics. The best material for such a tampon is sterilized gauze. The tampon may be employed in the impregnated and puerperal uterus, and also the unimpregnated uterus. In the impregnated uterus the intra-uterine application of the tampon may be made in preference to the vaginal application to bring on uterine contractions and empty the organ, or in some instances to ward off an abortion. It is of service in placenta previa, and is to be preferred to bougies, bags and tents for the purpose of inducing abortion in cases of uncontrollable vomiting or other serious conditions. During parturition, itmay be of service in assisting dilatation when the first stage is protracted. Post-partum it is useful in controlling hemorrhage; also for hemorrhage after an abortion, or for sepsis, after curettage and irrigation have been performed. In the unimpregnated uterus there is opportunity for its employment in causing dilatation for the purpose of exploring the uterine cavity in order to examine the nature and attachments of new growths. For the menorrhagia which frequently is seen in young women, associated with anemia, the use of the tampon, which makes direct pressure upon the vessels of the uterine cavity, is excellent. The many conditions of the endometrium which are associated with bleeding are improved by the use of the intra-uterine tampon packed firmly in the canal and up to the fundus, thus causing firm contraction of the uterine muscle and favoring free depletion. Thus it will be serviceable in endometritis, with hypertrophy or suppuration, in inertia of the uterus and stasis of the venous circulation. It is valuable after dilatation for stenosis and it may be helpful in effecting drainage of the Fallopian tube in a limited number of cases. As a rule it is better to have the patient in bed and at rest at the time of introducing the tampon, and in some cases an anesthetic may be necessary. It is quite safe and proper to introduce it in many cases at the office or dispensary, particularly if it is not passed much beyond the internal os. Dilatation, curettage and irrigation should usually precede its introduction, and the canal be dilated sufficiently to allow of the ready introduction of the gauze strips of which it is composed. The tampon may safely be retained from one to three days, although it is better to renew it frequently and secure gradual dilatation rather than pack in too much at first.—*Univ. Med. Mag.*

---

### THE CAUSATION OF THE DISEASES OF WOMEN.

*The American Gynecological Journal* for August, 1893, contains an abstract of a paper upon this subject by Charles P. Noble. The principal causes of the diseases of women are arranged under the following headings: (1) Improper development of the sexual organs. (2) Gonorrhea. (3) Septic inflammation following childbirth. (4) Lacerations due to childbirth. (5) Miscellaneous causes, including constipation, erroneous habits of life, and errors of dress. Under the first heading attention is called to the necessity of more careful advice from the physician regarding the establishment of normal menstruation at puberty. The temporary withdrawal from study or other taxing duties will enable girls of this age to acquire more perfect development of the sexual organs. Out-of-door exercise, nutritious food, and the use of tonics will greatly benefit them, and tend to prevent dysmenorrhea and sterility on the one hand, and lacerations and degenerations on the other.

Gonorrhea is one of the most important causes of the diseases of the uterus, tubes, ovaries and peritoneum. The exact percentage of cases of diseases of the appendages due to gonorrhea is not yet known.

Puerperal infection is a most frequent cause of disease, and this is considered by some as the most frequent cause of disease in the female sexual organs. The relative frequency of gonorrheal infection as compared with puerperal infection as a cause of inflammation of the uterus and its appendages is still a question in dispute. There is reason to believe that in some cases of inflammation following puerperal infection, the gonococcus may have been present in the parturient tract, and have caused the trouble, while the infection may have been attributed to other agents. The relations of lacerations of the cervix to subinvolution of the pelvic organs and of tears of the pelvic floor to rectocele, cystocele and prolapses of the uterus are well known. Constipation, constriction of the waist by the abuse of the corset, and the hanging of heavy skirts about the hips, together with the many unhygienic and enervating habits of women, contribute to cause disease of the sexual organs. In conclsion, the writer would call the attention of the general practitioner to the fact that the diseases of women are largely preventable, and that by careful oversight of the girl as she approaches the age of puberty; by vigorous treatment of gonorrhea and observation of the case until all abnormal discharges are arrested, and sexual intercourse prohibited until the disease is controlled; if antiseptic midwifery is practiced and lacerations are united promptly and attention be paid to the prevention of constipation, the ill effects of improper dressing and erroneous habits of living, the prevalence of the diseases peculiar to women would be greatly restricted.—*Univ. Med. Mag.*

